# Classification of recurrence status after surgical treatment of chronic subdural hemorrhage – A machine learning approach

**DOI:** 10.1371/journal.pone.0346756

**Published:** 2026-09-18

**Authors:** Hussam Hamou, Julius Kernbach, Hani Ridwan, Kimberley Fay-Rodrian, Hans Clusmann, Anke Hoellig, Michael Veldeman

**Affiliations:** 1 Department of Neurosurgery, RWTH Aachen University Hospital, Aachen, Germany; 2 Department of Neuroradiology, University Hospital Heidelberg, Heidelberg, Germany; 3 Department of Diagnostic and Interventional Neuroradiology, RWTH Aachen University, Aachen, Germany; Goethe University Hospital Frankfurt, GERMANY

## Abstract

**Background:**

Chronic subdural hematoma (cSDH) recurrence requiring reoperation occurs in 5–33% of cases. Predicting recurrence could enable risk-stratified surveillance, reducing imaging in low-risk patients while maintaining monitoring for high-risk individuals. We evaluated whether machine learning could achieve clinically actionable recurrence prediction using routinely available variables.

**Methods:**

This retrospective single-center study included 564 consecutive patients undergoing surgical cSDH evacuation (2015–2023), randomly divided into training (75%, n = 422) and test (25%, n = 142) sets. We developed and compared three models, regularized logistic regression, Random Forest, and XGBoost, using 31 predictor variables. Model development and tuning used 10-fold cross-validation on the training set; the best model was evaluated on the held-out test set. The primary outcome was postoperative recurrence requiring reoperation.

**Results:**

Postoperative recurrence occurred in 170 patients (30.1%). In the training set, XGBoost achieved the highest cross-validated ROC AUC (0.713, SE = 0.024), matching Random Forest and outperforming logistic regression (0.686). Hematoma volume, coagulation parameters, and disease severity markers (ICU admission, GCS) were the most influential predictors, though effect sizes remained modest. On the test set, the final XGBoost model achieved ROC AUC 0.688 (95% CI 0.590–0.772), with satisfactory average calibration but overconfident individual-level risk estimates (calibration slope 0.615). At the clinically relevant 90% sensitivity threshold, specificity was only 30.3%, allowing potential imaging reduction in roughly one-third of non-recurrence patients. Consistency between training and test performance indicated these limitations reflect predictor information content rather than overfitting.

**Conclusions:**

In this single-center cohort, machine learning models using routinely available clinical and radiographic variables did not achieve clinically actionable risk stratification for cSDH recurrence under internal validation, with discriminative capacity insufficient to identify a low-risk subgroup suitable for de-escalated surveillance. These findings suggest recurrence is driven by factors not captured in standard clinical assessment, supporting uniform or symptom-driven imaging strategies over risk-stratified approaches.

## Introduction

Chronic subdural hematoma (cSDH) has an estimated annual incidence ranging from 7 to 30 per 100,000 individuals in population-based surveys [1–3]. The incidence rises markedly with advancing age, reaching 58 per 100,000 in patients over 70 years old [4]. Driven by population aging and increased use of antithrombotic medications, the global burden of cSDH continues to rise, with corresponding increases in healthcare costs and resources used [5].

The pathophysiology of cSDH involves a complex cascade of events typically believed to be initiated by minor head trauma, causing either rupture of a bridging vein or traumatic separation of the dural border cell layer [6,7]. In susceptible individuals, the initial subdural collection fails to resorb. Instead, it undergoes liquefaction and gradual expansion through a self-perpetuating cycle of inflammation, neoangiogenesis, and recurrent hemorrhage from fragile neomembranes [6,7].

Surgical evacuation, typically performed via burr hole craniotomy with subdural drainage, remains the treatment of choice for symptomatic hematomas [8]. However, postoperative recurrence necessitating reoperation occurs in 5–33% of cases, representing a substantial clinical and economic burden [9]. Identified risk factors for recurrence include larger hematoma volume, ongoing antithrombotic treatment, history of epileptic seizures, incomplete brain re-expansion, and less organized hematoma architecture on computed tomography (CT) imaging [9,10]. Though there are good arguments in favor of only performing follow-up scans in patients with recurring symptoms [11], current practice in many centers still involves imaging for all patients following surgical evacuation. This results in cumulative healthcare expenditure, which may be a consequence of overcautious practice.

The ability to accurately predict which patients are at highest risk for recurrence could enable risk-stratified follow-up protocols, potentially reducing unnecessary imaging in low-risk patients while maintaining close surveillance for those at high risk. However, despite numerous studies identifying individual risk factors [9], no validated clinical prediction model exists to guide personalized follow-up strategies. Traditional statistical approaches have yielded models with limited discriminative capacity, possibly reflecting the complex, non-linear relationships between clinical variables and recurrence risk that exceed the capabilities of conventional regression methods [12–15].

Machine learning algorithms, including ensemble methods such as Random Forest and gradient boosting, have demonstrated superior performance in clinical prediction tasks by capturing non-linear relationships and complex variable interactions without requiring explicit specification [16]. These methods have been successfully applied to predict outcomes in various neurosurgical conditions, yet their utility for cSDH recurrence prediction remains underexplored. If sufficiently accurate, such models could identify a low-risk patient subgroup suitable for de-escalated surveillance protocols, balancing the clinical imperative to detect recurrences against the harms of repeated radiation exposure and the resulting additional costs.

The primary objective of this study was to develop and validate machine learning models to predict postoperative cSDH recurrence using variables available during the perioperative time window, incl. clinical, demographic, laboratory, and radiographic variables. Secondary objectives included identifying the most influential predictors of recurrence through variable importance analysis.

## Methods

### Study population and design

All consecutive patients who underwent surgical treatment for chronic subdural hematoma at a single tertiary care university hospital (RWTH Aachen University Hospital, Aachen, Germany) between January 2015 and December 2023 were considered for inclusion. This study represents an extension of previously published cohorts from the same institution [10,17,18], with the current analysis focusing on machine learning-based prediction of postoperative recurrence. This research was performed in accordance with all relevant guidelines and regulations, and in accordance with the Declaration of Helsinki. The study was approved by the local ethics committee (1EK 25/331) and registered in the German Clinical Trials Register (DRKS00025280). Informed consent was waived due to the retrospective design. This manuscript is written in accordance with the Guidelines for Developing and Reporting Machine Learning Predictive Models in Biomedical Research [19].

Inclusion criteria comprised all patients with chronic subdural hematoma confirmed on computed tomography (CT) imaging who underwent surgical evacuation via twist drill craniostomy, burr hole craniotomy, or bone flap craniotomy. Patients were excluded if: (1) they were under 18 years of age; (2) initial CT imaging was unavailable or not uploaded to the institutional picture archiving and communication system; (3) intracranial hypotension (e.g., shunt overdrainage or spinal cerebrospinal fluid leak) contributed to hematoma formation; (4) prior neurosurgical or other cranial procedures were causally related to hematoma development; or (5) non-iatrogenic coagulation disorders (e.g., hepatogenic coagulopathy or bleeding diathesis) were present.

### Data collection

Data were collected retrospectively through a systematic review of electronic hospital records. Identifiable hospital records were accessed for research purposes by HH, JK, HR, KR, AH, and MV between 12/09/2025 and 31/12/2025. All named investigators are physicians who were directly involved in the clinical care of the included patients at the Department of Neurosurgery, University Hospital RWTH Aachen, and therefore have legitimate and continuous access to these medical records as required for patient treatment and follow-up. The data access dates reported reflect the period during which clinical records were reviewed for the purpose of data extraction, rather than the granting of new or exclusive research access. Extracted variables included patient demographics (age, sex), documented history of head trauma, presenting clinical symptoms and neurological deficits (paresis, gait disturbance, speech disorder, reduced level of consciousness, headache, nausea, vertigo, personality changes), Glasgow Coma Scale (GCS) score at admission, pre-existing medical comorbidities (hypertension, coronary artery disease, cardiac arrhythmias, diabetes mellitus, pulmonary disease, kidney disease, cancerous disease, prior neurological disease, alcohol abuse, prior seizure or epilepsy, peripheral arterial disease, prior deep venous thrombosis or pulmonary embolism, prior myocardial infarction or stroke, hematologic or hepatic disease), pre-admission medication use (antiplatelet therapy, dual antiplatelet therapy, oral anticoagulation including vitamin K antagonists and direct oral anticoagulants, angiotensin-converting enzyme inhibitors, angiotensin receptor blockers, statins), American Society of Anesthesiologists (ASA) physical status classification, pre-operative laboratory values (activated partial thromboplastin time, international normalized ratio, platelet count), and indication for emergency surgery.

Perioperative variables included surgical technique employed, pre-operative coagulation optimization (administration of vitamin K, prothrombin complex concentrate, thrombocyte concentrate, fresh frozen plasma, or tranexamic acid), post-operative anti-thrombotic management, use of early mobilization protocols, and post-operative need for intensive care observation. Primary outcome variables comprised of postoperative recurrence requiring reoperation after initial patients’ discharge.

### Treatment algorithm

Surgical evacuation was indicated for patients presenting with neurological deficits (paresis, gait disturbance, speech disorder, or seizures) or for asymptomatic patients with radiographic evidence of mass effect (midline shift, ventricular compression, or sulcal effacement). An isolated headache without objective neurological findings or mass effect was not considered an indication for surgery.

The primary surgical approach was burr hole craniotomy with irrigation and placement of one or two non-suction subdural silicon drains (12-French) under general anesthesia or conscious sedation. In cases demonstrating intraoperative brain expansion with limited subdural space, a single drain was placed. Drains were typically removed after 1–3 days once drainage ceased. Twist drill craniostomy under local anesthesia without irrigation was reserved for homogenous hematomas in medically frail patients. Bone flap craniotomy was considered when pre-operative imaging demonstrated hyperdense clot components, suggesting complete evacuation via a burr hole approach would be inadequate. Visceral membranes were opened only if encapsulated deeper hematoma compartments were suspected; otherwise, membranous structures were left intact. The choice of surgical technique was left to the operating surgeon’s discretion. No included patients received middle meningeal Artery embolization.

Pre-operative antihypertensive medications, including angiotensin-converting enzyme inhibitors and angiotensin receptor blockers, were not routinely discontinued prior to surgery. Pre-operative coagulation optimization was performed as clinically indicated based on anticoagulation status and laboratory values.

### Postoperative management and follow-up protocol

Postoperative care was standardized regardless of the surgical approach, with drain removal on postoperative day 2 or 3 for patients undergoing burr hole craniotomy. Routine postoperative imaging during the initial hospitalization was performed for twist drill craniostomy cases and selectively for burr hole craniotomy patients who developed new symptoms, experienced persistent pre-operative deficits, or had clinical concern for inadequate evacuation. Identification of a significant residual hematoma with mass effect triggered revision surgery during the same admission. The surgical technique recorded for each patient corresponds to the final procedure performed during the index admission; patients whose initial twist drill craniostomy was converted to burr hole craniotomy within the same hospital stay due to inadequate evacuation were therefore classified as having undergone burr hole craniotomy.

Patients were discharged when complete symptom resolution was achieved or when postoperative imaging demonstrated resolution of the space-occupying effect. After discharge, surveillance CT imaging was performed 14–28 days post-surgery and continued at 2- to 3-week intervals until radiological resolution was documented. Asymptomatic residual hematomas without mass effect were monitored with sequential imaging until either complete resolution or clinical progression occurred. Small persistent asymptomatic subdural fluid collections without mass effect in elderly patients with brain atrophy were considered resolved, and surveillance was discontinued.

### Definition of recurrence

Postoperative recurrence was defined as an increase in volume of residual or newly formed subdural hematoma that demonstrated mass effect (midline shift, sulcal effacement, or ventricular compression) or caused new onset or recurrence of neurological symptoms (paresis, gait disturbance, speech disorder, or reduced level of consciousness) necessitating reoperation. No absolute volume threshold was applied; clinical judgment incorporating both radiographic and clinical factors determined the need for surgical revision. Hematoma recurrence occurring more than 6 months after initial treatment, with documented complete resolution in the interim, was considered new disease rather than recurrence and was excluded from analysis. By definition, reoperation performed during the index admission for inadequate primary evacuation was not classified as recurrence; only reoperation occurring after discharge was counted as the primary outcome. The 6-month cutoff was chosen to align with the follow-up horizon commonly used as a primary endpoint window in contemporary cSDH intervention trials, while accommodating our institution’s practice of continuing surveillance until radiological resolution was confirmed, which for the large majority of patients occurred well within this window. No consensus definition of the recurrence-versus-new-disease distinction exists across the cSDH literature, with published windows ranging from 4–12 weeks to 6 months [9].

### Radiological assessment

Pre-operative hematoma dimensions were measured on axial CT images, including maximum length along the longest axis (mm), maximum width (mm), and total volume (mL) using software-assisted three-dimensional reconstruction (Brainlab, Munich, Germany). For bilateral hematomas, measurements of the larger hematoma were used for unilateral dimensional analysis (e.g., max. width), while in addition, the total bilateral volume was calculated. This mixed approach reflects clinical practice: maximal unilateral dimensions capture the size of the dominant lesion most likely driving mass effect and symptoms, while total bilateral volume reflects the cumulative burden motivating surgical decision-making. Laterality itself (unilateral versus bilateral) was included as a separate predictor variable in all models. The presence and extent of midline shift (mm) were documented; blinding status was not separately recorded for volumetric and midline shift measurements, in contrast to architecture classification, which was performed with blinding to clinical outcome.

Each hematoma was classified according to internal architecture on CT imaging into one of eight subtypes by two independent assessors (HH and MV), blinded to clinical outcomes, as previously described [10]. Interrater reliability for this classification system was previously quantified in an overlapping cohort using the same two assessors, yielding a Cohen’s kappa of 0.835 (p < 0.001) for the eight-category extended classification, indicating almost perfect agreement. The classification system comprised: (1) homogenous hypodense, appearing hypodense relative to brain parenchyma; (2) homogenous isodense, appearing isodense to brain tissue; (3) homogenous hyperdense, appearing hyperdense without recent acute trauma; (4) sedimented, with visible layering of hemoglobin sediment separated by gravity; (5) laminar, with visible hyperdense visceral or parietal membrane; (6) bridging, with countable internal membranes connecting visceral and parietal surfaces; (7) trabecular, with complex diffuse membrane architecture precluding individual membrane counting; and (8) subacute, with acute blood components admixed within chronic hematoma in the absence of recent trauma. When features of multiple subtypes coexisted, hematomas were classified according to the most organized subtype present.

For the machine learning analysis, the eight-category architectural classification was collapsed into a simplified four-category system: homogeneous (combining hypodense, isodense, and hyperdense subtypes), organized (combining laminar, bridging, and trabecular subtypes), sedimented, and subacute. As a sensitivity check, repeating all three models using the original eight-category classification in place of this collapsed four-category version yielded materially unchanged cross-validated performance (Δ AUC ≤ 0.006 for all models) and test-set discrimination consistent with split-to-split sampling variability (Results), confirming that the four-category simplification did not meaningfully reduce predictive information.

### Data preparation and missing data handling

All statistical analyses and visualizations were conducted using R (v4.4.0; www.r-project.org) within RStudio (v2024.12.0 + 467). The tidyverse was used for data processing along the tidymodels framework for model development and evaluation.

Of the 630 patients in the initial cohort, 564 patients (89.5%) had complete data on the primary outcome variable (recurrence status) and were included in the analysis. Baseline characteristics were compared between included and excluded patients to assess potential selection bias. An overview of these analyses is presented in Table 1. The final analytic dataset comprised 31 predictor variables, including patient demographics, clinical presentation, comorbidities, medication use, laboratory values, hematoma characteristics, and postoperative features. Missing data in predictor variables ranged from 0% to 4.8%, with no single variable exceeding 5% missingness. To maximize statistical power and avoid loss of information from complete case analysis, missing predictor values were imputed using median imputation for continuous variables and mode imputation for categorical variables. Imputation was performed within each cross-validation fold to prevent data leakage. For the held-out test set, imputation values (medians for continuous variables, modes for categorical variables) were derived exclusively from the training set and applied without modification, preventing information leakage from the test set into imputed values. Postoperative residual hematoma volume and calculated brain re-expansion, though recorded when available, were not included as model predictors: because immediate postoperative imaging was performed routinely only for twist drill craniostomy and selectively for burr hole or bone flap craniotomy patients with new or persistent symptoms, postoperative volume was missing in 50.0% of the analytic cohort overall, with missingness highest among burr hole craniotomy patients (60.8%). Because imaging ascertainment was determined by clinical status rather than occurring at random, this missingness is informative rather than at random (MNAR), and neither multiple imputation nor restriction to the subset with available imaging was considered appropriate; these variables were therefore excluded from model development.

**Table 1:** Demographic, clinical and hematoma specific parameters.

Parameter	All	No recurrence	Recurrence	p-value
— Demographics —	564	394 (69.9%)	170 (30.1%)	
Age (years)	78.0 [69.0–83.0]	78.0 [68.2–84.0]	77.0 [70.0–83.0]	0.704
sex (f/m)	195/369 (34.6%/65.4%)	135/259 (34.3%/65.7%)	60/110 (35.3%/64.7%)	0.813
Trauma history	410 (72.7%)	288 (73.1%)	122 (71.8%)	0.745
Cardiac arrhythmia	126 (22.4%)	90 (22.9%)	36 (21.3%)	0.677
Hypertension	349 (62.0%)	252 (64.1%)	97 (57.1%)	0.113
Coronary artery disease	169 (30.0%)	111 (28.2%)	58 (34.1%)	0.163
Diabetes	100 (17.8%)	74 (18.8%)	26 (15.3%)	0.314
Cancer	73 (13.0%)	47 (12.0%)	26 (15.4%)	0.268
Alcohol abuse	25 (4.4%)	17 (4.3%)	8 (4.7%)	0.830
Peripheral arterial disease	88 (15.7%)	63 (16.0%)	25 (14.8%)	0.711
ACE inhibitor	196 (34.8%)	133 (33.8%)	63 (37.1%)	0.450
Sartan	94 (16.7%)	68 (17.3%)	26 (15.3%)	0.566
Statin	178 (31.6%)	131 (33.3%)	47 (27.6%)	0.183
Antiplatelet therapy	184 (32.7%)	136 (34.6%)	48 (28.2%)	0.139
Oral anticoagulation	90 (16.0%)	69 (17.6%)	21 (12.4%)	0.119
Pre-op aPTT	28.9 [26.8–31.0]	28.9 [26.9–31.3]	28.6 [26.6–30.7]	0.229
Pre-op INR	1.1 [1.0–1.1]	1.1 [1.0–1.2]	1.0 [1.0–1.1]	0.002
Pre-op thrombocytes	232.0 [190.0–287.0]	231.5 [187.0–293.2]	239.0 [193.0–277.0]	0.765
— Clinical presentation —				
GCS at admission	15.0 [14.0–15.0]	15.0 [14.0–15.0]	15.0 [14.0–15.0]	0.311
Emergency surgery	115 (20.4%)	98 (24.9%)	17 (10.0%)	<0.001
Pre-op epilepsy	50 (8.9%)	34 (8.7%)	16 (9.5%)	0.755
Pre-op paresis	272 (48.7%)	184 (46.9%)	88 (52.7%)	0.213
Pre-op reduced consciousness	150 (27.9%)	100 (26.2%)	50 (32.1%)	0.173
Pre-op phasic disorder	132 (23.6%)	100 (25.5%)	32 (19.2%)	0.106
Missing GCS	2 (0.4%)	2 (0.5%)	0 (0.0%)	
— Hematoma characteristics —				
Laterality: unilateral	413 (73.2%)	292 (74.1%)	121 (71.2%)	0.470
Laterality: bilateral	151 (26.8%)	102 (25.9%)	49 (28.8%)	0.470
Hematoma volume (mL)	131.5 [98.2–169.8]	127.0 [90.0–168.0]	143.5 [112.2–176.0]	0.004
Midline shift present	398 (72.8%)	272 (72.0%)	126 (74.6%)	0.528
Hematoma type (collapsed) (overall)				<0.001
homogeneous	229 (41.1%)	130 (33.5%)	99 (58.6%)	
organized	222 (39.9%)	179 (46.1%)	43 (25.4%)	
sedimented	33 (5.9%)	17 (4.4%)	16 (9.5%)	
subacute	73 (13.1%)	62 (16.0%)	11 (6.5%)	
Missing volume	6 (1.1%)	6 (1.5%)	0 (0.0%)	
Missing hematoma type	7 (1.2%)	6 (1.5%)	1 (0.6%)	
Surgical technique (overall)				<0.001
Twist drill craniostomy	178 (31.6%)	57 (14.5%)	121 (71.2%)	
Burr hole craniotomy	372 (66.0%)	324 (82.2%)	48 (28.2%)	
Bone flap craniotomy	14 (2.5%)	13 (3.3%)	1 (0.6%)	
Missing surgical technique	0 (0.0%)	0 (0.0%)	0 (0.0%)	
— Post-operative features —				
Post-op paresis	66 (11.8%)	44 (11.3%)	22 (13.0%)	0.552
Post-op reduced consciousness	45 (8.1%)	31 (8.0%)	14 (8.2%)	0.929
ICU admission	340 (60.5%)	207 (52.8%)	133 (78.2%)	<0.001

### Model development strategy

The dataset was randomly partitioned into training (75%, n = 422) and test (25%, n = 142) sets using stratified sampling to maintain the outcome class distribution (approximately 30% recurrence rate) in both subsets. All model development and hyperparameter tuning were performed exclusively on the training set using 10-fold stratified cross-validation. The holdout test set was reserved for final model evaluation.

Predictor variables were preprocessed prior to model fitting. Continuous variables were standardized to have mean zero and unit variance. Categorical variables were encoded using dummy variables with reference category coding. Zero-variance predictors were identified and removed if present.

### Regularized logistic regression

We first developed a regularized logistic regression model using elastic net regularization, which combines L1 (lasso) and L2 (ridge) penalties to handle correlated predictors and perform automatic feature selection. Penalty strength and mixing parameter were tuned via grid search (50 combinations; full grid specification in Supplementary Methods S1 Table) across all cross-validation folds.

### Performance metrics

Model performance was evaluated using multiple metrics appropriate for binary classification with mild class imbalance. The primary performance metric was the area under the receiver operating characteristic curve (ROC AUC). Secondary metrics included overall accuracy, sensitivity (recall), specificity, positive predictive value (precision), and negative predictive value. All metrics were calculated from cross-validation predictions and reported as means with standard errors across the 10 folds.

### Clinical decision context and threshold selection

The primary clinical objective was to identify patients at low risk of recurrence who could safely undergo less intensive follow-up. In this context, false positive predictions (classifying low-risk patients as high-risk) are clinically acceptable, as these patients would continue standard surveillance. Conversely, false negative predictions (failing to identify patients who develop recurrence) carry greater clinical risk, as these patients might be inappropriately allocated to reduced surveillance and experience delayed detection of recurrence. Therefore, model threshold selection prioritized high sensitivity (minimizing false negatives) over specificity. We considered sensitivity thresholds of 85%, 90%, and 95% as clinically relevant benchmarks, with the understanding that higher sensitivity values would necessarily result in lower specificity and fewer patients eligible for reduced imaging. The optimal threshold was selected to achieve at least 90% sensitivity while maximizing specificity, balancing the clinical imperative to detect recurrences against the potential to reduce unnecessary imaging in a subset of low-risk patients. All predictor variables, including postoperative features such as ICU admission and postoperative neurological status, were ascertained during the index hospital admission and were therefore available in full by the time of discharge; the model is accordingly intended to be applied at the point of discharge planning to stratify patients according to their predicted risk of post-discharge recurrence, consistent with our outcome definition, which counts only recurrence occurring after discharge.

### Random Forest and XGBoost models

To explore whether ensemble machine learning methods could improve upon the performance of regularized logistic regression, we developed Random Forest and XGBoost models. We constructed a Random Forest model with 500 trees, tuning the number of predictors sampled at each split (mtry) and minimum node size (min_n) via grid search within the 10-fold cross-validation framework (30 combinations; full grid in Supplementary Methods S1 Table), selecting the configuration with the highest mean ROC AUC. Permutation-based variable importance was calculated on the final model by randomly shuffling each predictor variable and measuring the resulting decrease in ROC AUC, averaged across all cross-validation folds.

XGBoost was implemented as an alternative ensemble approach. Categorical predictors were dummy-coded prior to model fitting. We trained XGBoost models with 500 boosting rounds, tuning maximum tree depth, learning rate, and minimum node size via the same cross-validation framework (36 combinations; Supplementary Methods S1 Table).

Both ensemble methods used the same imputation strategy as logistic regression, with median imputation for continuous variables and mode imputation for categorical variables applied within each cross-validation fold. Performance was compared across all three modeling approaches using ROC AUC as the primary metric, with clinical utility evaluated through threshold analysis at sensitivity targets of 85%, 90%, and 95%.

### Variable importance and model interpretability analysis

To understand which features contributed most to recurrence prediction and how they influenced model predictions, we conducted comprehensive interpretability analyses on the best-performing model. Variable importance was quantified using permutation-based importance scores, which measure the decrease in model performance when each feature’s values are randomly shuffled. This approach provides a model-agnostic assessment of feature relevance that accounts for potential interactions.

To elucidate the direction and magnitude of individual feature effects, we computed SHapley Additive exPlanations (SHAP) values. We calculated SHAP values for all features using 50 Monte Carlo samples per observation to balance computational feasibility with estimate stability. SHAP summary plots were generated to visualize both the magnitude of feature effects (mean absolute SHAP value) and the directionality of these effects as a function of feature values.

Partial dependence plots were constructed for the top continuous predictors to illustrate the marginal effect of each feature on predicted recurrence probability while averaging over the effects of all other features.

### Univariate statistics

Descriptive data are presented as mean and standard deviation (SD) for normally and as median and interquartile range (IQR: Q_1_ to Q_3_) for nonnormally distributed continuous variables. Categorical data are provided as proportions (%). After normality testing by means of the Shapiro-Wilk test, the appropriate statistical test was selected. For normally distributed continuous data, the unpaired t-Test and for nonnormally distributed data the Mann-Whitney U-Test was applied. For nominal data, the χ^2^ -Test was used. An alpha level was fixed at 0.05.

## Results

### Study population

**Fig 1:**
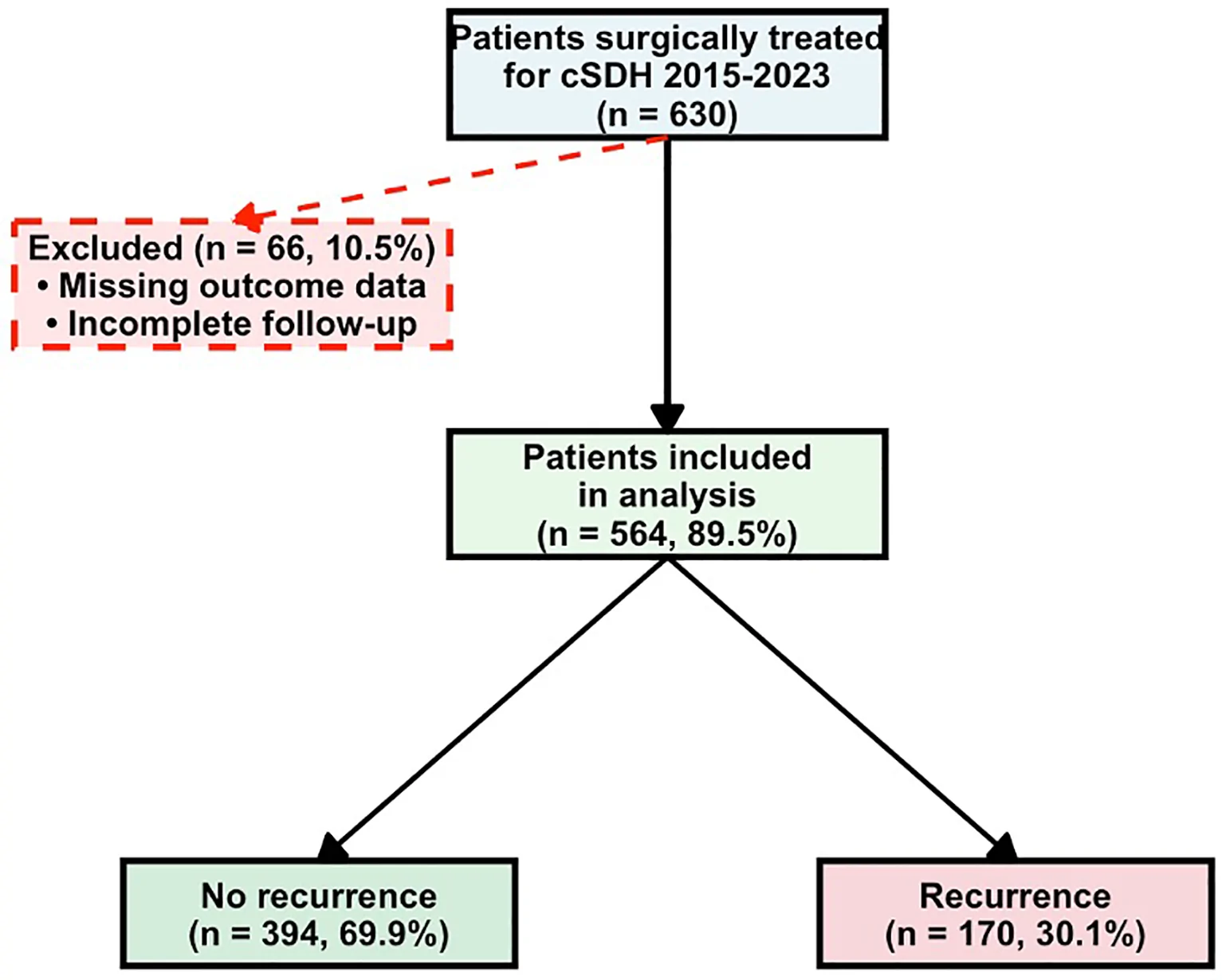
Inclusion flow chart. Flow diagram illustrating patient selection for the machine learning analysis of chronic subdural hematoma (cSDH) recurrence prediction. Of 630 consecutive patients who underwent surgical treatment for cSDH at a single tertiary care center between January 2015 and December 2023, 66 patients (10.5%) were excluded due to missing outcome data or incomplete follow-up, resulting in a final analytic cohort of 564 patients (89.5%). Among included patients, 170 (30.1%) experienced postoperative recurrence requiring reoperation, while 394 (69.9%) did not develop recurrence during follow-up. This class imbalance ratio of 2.32:1 (no recurrence to recurrence) was maintained in the stratified train-test split for model development and validation.

Between January 2015 and December 2023, a total of 630 consecutive patients underwent surgical treatment for chronic subdural hematoma at our institution. After excluding 66 patients (10.5%) with missing recurrence outcome data due to incomplete follow-up or unavailable imaging records, 564 patients (89.5%) constituted the final analytic cohort. Excluded and included patients did not differ significantly in mortality: among excluded patients with documented mortality status (33 of 66, 50.0%), none died during the observation period, compared with 3.2% (18 of 561) among included patients with documented status (Fisher’s exact test, p = 0.616). Mortality status was itself missing in half of excluded patients, consistent with the broader pattern of incomplete documentation or loss to follow-up that led to their exclusion, rather than indicating a differential mortality-related selection process. The inclusion process is depicted as a flow-chart (Fig 1).

The complete cohort was randomly divided into a training set (n = 422, 75%) for model development and hyperparameter tuning, and a held-out test set (n = 142, 25%) for final model evaluation. The median age was 78 years (interquartile range 69–83 years), and 369 patients (65.4%) were male. A documented history of head trauma was present in 410 patients (72.7%). Pre-operative antithrombotic therapy was common, with 184 patients (32.7%) receiving antiplatelet agents and 90 patients (16.0%) receiving oral anticoagulation. The most frequently observed comorbidities included hypertension (62.0%), coronary artery disease (30.0%), and cardiac arrhythmias (22.4%).

Postoperative recurrence requiring reoperation occurred in 170 patients (30.1%), while 394 patients (69.9%) did not experience recurrence during follow-up. The recurrence rate of 30.1% represents a class imbalance ratio of 2.32:1 (no recurrence to recurrence). Baseline characteristics stratified by recurrence status are presented in Table 1. Patients who experienced recurrence had significantly larger pre-operative hematoma volumes (median 143.5 mL vs 127.0 mL, p = 0.004), lower rates of emergency surgery (10.0% vs 24.9%, p < 0.001), higher rates of ICU admission postoperatively (78.2% vs 52.8%, p < 0.001), and different distributions of hematoma architectural subtypes (p < 0.001). Homogeneous hematomas were more prevalent in the recurrence group (58.6% vs 33.5%), while organized hematomas were more common in patients without recurrence (46.1% vs 25.4%). Pre-operative INR values were slightly but significantly lower in patients who developed recurrence (1.0 vs 1.1, p = 0.002). No significant differences were observed between groups regarding age, sex, trauma history, comorbidities, or most medication use.

### Model development and cross-validation performance (training set)

All model development, hyperparameter tuning, and performance comparisons described in this section were conducted exclusively on the training set (n = 422) using 10-fold stratified cross-validation. The held-out test set (n = 142) was not used during model development and was reserved solely for final evaluation of the selected model.

### Model performance comparison (training set)

**Fig 2:**
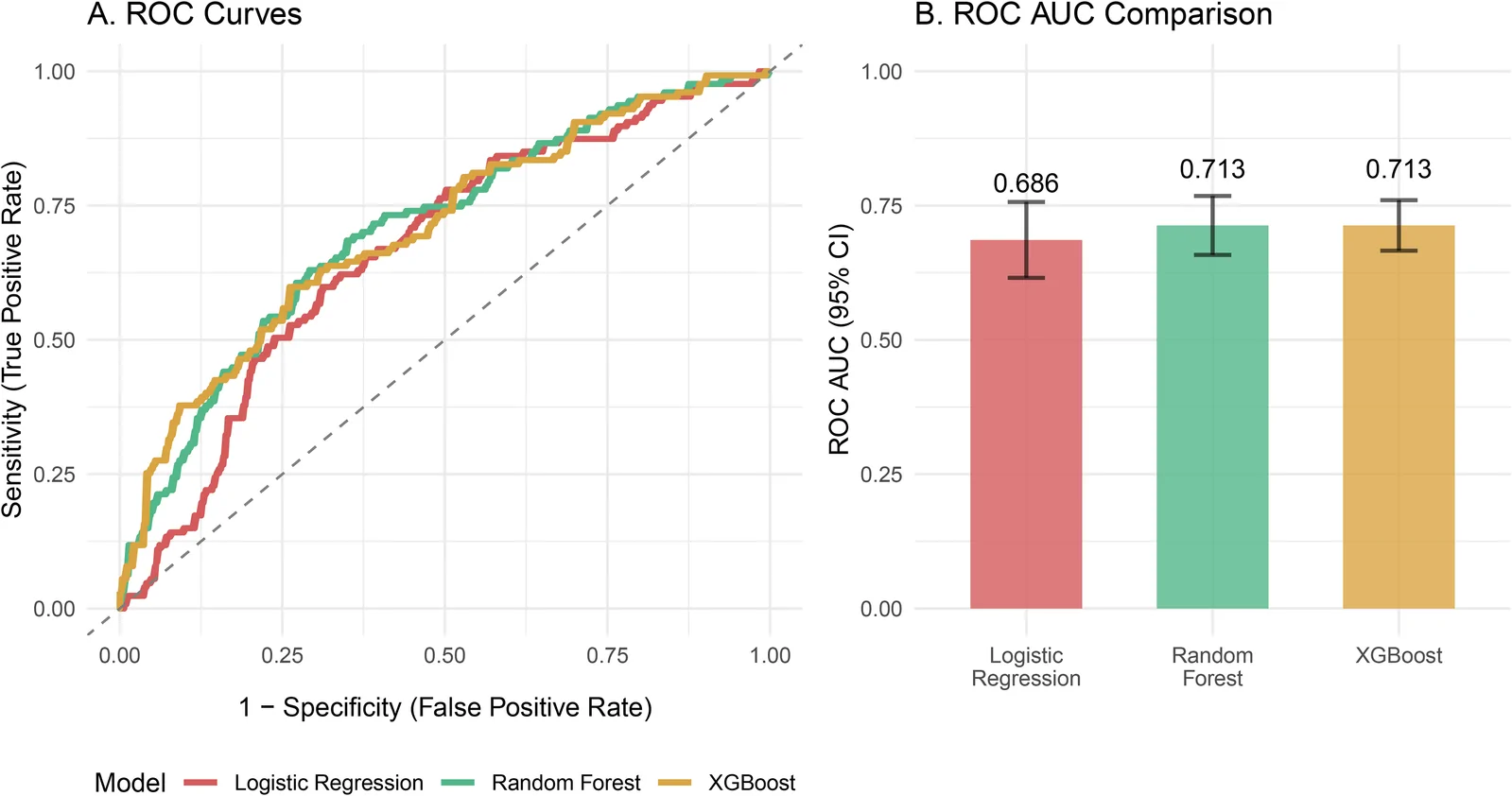
Comparison of machine learning model performance for predicting chronic subdural hematoma recurrence. (A) Receiver Operating Characteristic (ROC) curves comparing the discriminative performance of three machine learning algorithms: regularized logistic regression (red), Random Forest (green), and XGBoost (blue). All models were evaluated using 10-fold stratified cross-validation on the training dataset (n = 422 patients). The diagonal dashed line represents the performance of a random classifier (AUC = 0.5). Curves positioned above the diagonal indicate better-than-chance discrimination between patients who developed recurrence and those who did not. (B) Area under the ROC curve (AUC) values with 95% confidence intervals for each model. Error bars represent 95% confidence intervals calculated from cross-validation standard errors. Both ensemble methods (Random Forest and XGBoost) achieved identical AUC values of 0.713, demonstrating modest but statistically significant improvement over regularized logistic regression (AUC = 0.686). Despite the superior discrimination metrics of ensemble approaches, all three models converged to similar performance levels, suggesting a ceiling effect determined by the inherent predictive capacity of the available clinical, demographic, laboratory, and radiographic features rather than algorithmic sophistication. The modest AUC values (0.68–0.71) indicate limited discriminative ability, corresponding to the study’s finding that these models cannot achieve clinically actionable risk stratification for surveillance imaging protocols.

We evaluated three modeling approaches: regularized logistic regression (elastic net), Random Forest, and XGBoost, each tuned via 10-fold cross-validation on the training set. Optimal configurations were: logistic regression, penalty 0.053, mixture 0.75; Random Forest, mtry 12, min_n 11; XGBoost, tree depth 5, learning rate 0.01, min_n 5. Logistic regression achieved a mean cross-validated ROC AUC of 0.686 (SE 0.036), while both ensemble methods reached an identical, modestly higher AUC of 0.713 (Random Forest SE 0.028; XGBoost SE 0.024), a small but statistically discernible improvement attributable to their capacity to model non-linear relationships and variable interactions (see Fig 2). Absolute risk separation nonetheless remained limited across all three models: mean predicted probabilities for patients with vs. without recurrence were 0.333 vs. 0.287 (logistic regression), 0.387 vs. 0.276 (Random Forest), and 0.403 vs. 0.241 (XGBoost), with XGBoost showing the widest predicted-probability range (0.04–0.91) and the lowest cross-validation variance.

**Fig 3:**
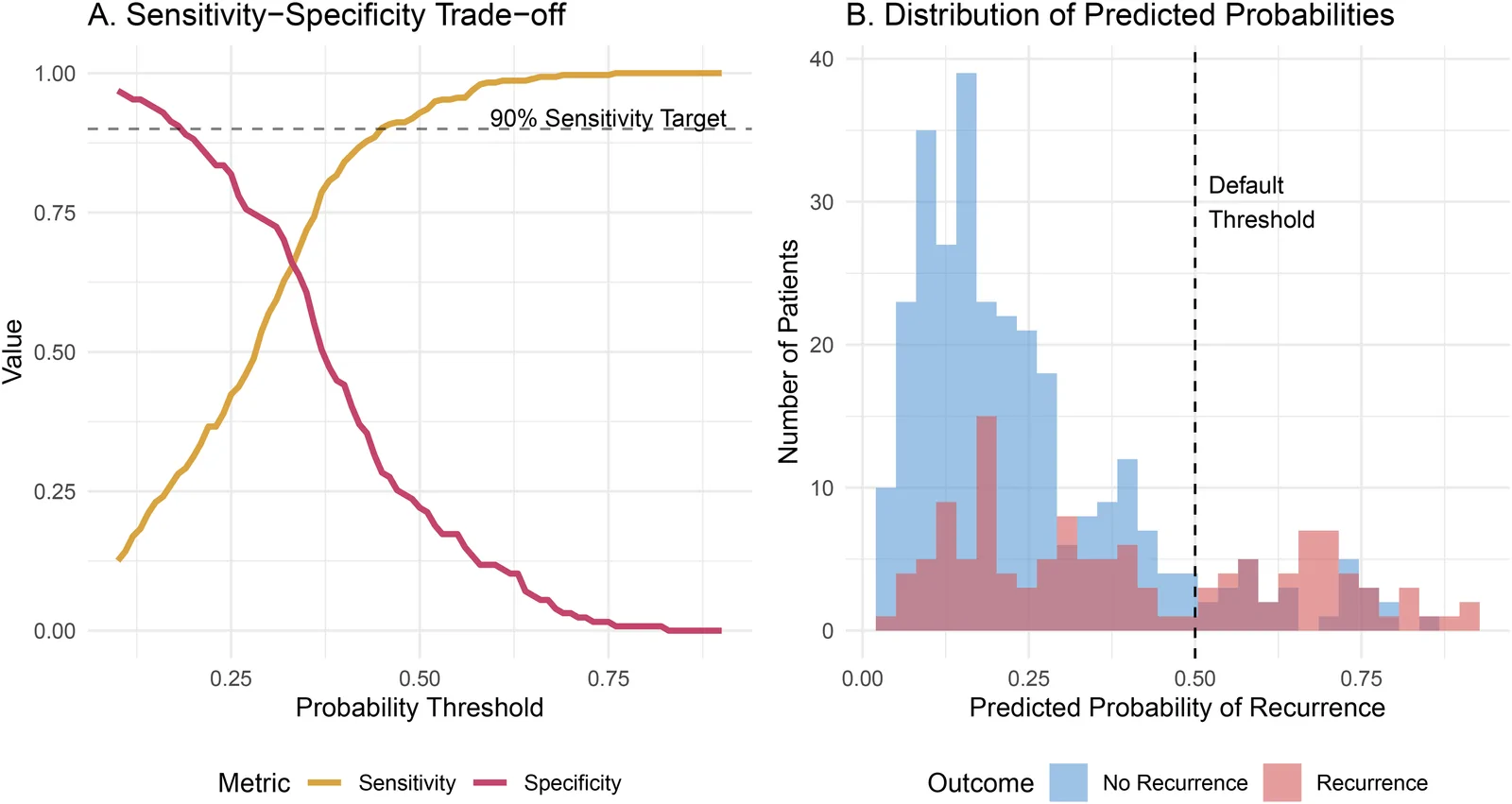
Clinical utility analysis and predicted probability distributions. (A) Sensitivity-Specificity Trade-off Curve illustrating the inverse relationship between sensitivity (green) and specificity (red) across different probability thresholds for the most competitive (XGBoost) model. The horizontal dashed line indicates the clinically relevant 90% sensitivity target, representing the minimum acceptable threshold for safe de-escalation of surveillance imaging. At this sensitivity level, the model achieves near-zero specificity, demonstrating its inability to identify a meaningful low-risk patient subgroup. The steep decline in specificity as sensitivity increases reflects the compressed range of predicted probabilities and limited discriminative capacity of the model. (B) Distribution of Predicted Probabilities showing the overlap between patients who did not experience recurrence (yellow, n = 394) and those who developed recurrence (red, n = 170) based on XGBoost model predictions from 10-fold cross-validation. The vertical dashed line represents the default classification threshold of 0.5. Substantial overlap between the two distributions, particularly in the 0.2–0.4 probability range where most predictions cluster, illustrates why threshold optimization cannot achieve clinically actionable risk stratification. Only a small proportion of patients receive high-confidence predictions (probabilities >0.6 or <0.1), precluding reliable identification of patients suitable for reduced surveillance protocols. The distribution pattern provides visual evidence for the model’s limited clinical utility despite statistically significant discriminative performance (AUC 0.713).

Threshold analysis prioritizing 90% sensitivity, necessary to avoid missing recurrences, showed near-zero specificity for logistic regression and Random Forest, and only 1.6% specificity for XGBoost (5 of 295 non-recurrence patients), indicating that none of the three approaches could identify a clinically useful low-risk subgroup. Results of the clinical utility analysis for XGBoost, the best-performing and most stable model, are depicted in Fig 3. The convergence of three fundamentally different modeling paradigms (regularized linear regression, bootstrap aggregation, and gradient boosting) to nearly identical discrimination (AUC approximately 0.71) and clinical utility provides strong evidence that the current predictor set had reached its ceiling of achievable performance, independent of algorithmic sophistication. Full per-model results, including detailed predicted-probability distributions and threshold curves for logistic regression and Random Forest individually, are provided in Supplementary Results (S2,S3 Tables, S1 Fig.).

The convergence of three fundamentally different modeling approaches (linear regression with regularization, bootstrap aggregation, and gradient boosting) to nearly identical cross-validated ROC AUC values of approximately 0.71 within the training set provides strong evidence that maximal discriminative performance has been achieved with the current predictor set.

### Rationale for modeling approach

We evaluated three fundamentally distinct machine learning paradigms: regularized logistic regression, Random Forest, and XGBoost. The near-identical cross-validated ROC AUC values achieved by Random Forest (0.713) and XGBoost (0.713) within the training set, combined with their similar clinical utility profiles at high sensitivity thresholds, suggested that these ensemble methods had extracted maximal predictive information from the available feature set.

Additional linear discriminant-based approaches such as LDA and QDA were not pursued, as these methods assume multivariate normality and would be expected to perform similarly to or worse than regularized logistic regression, which makes fewer distributional assumptions while accommodating the mixed continuous and categorical nature of our predictors. Naive Bayes classification was similarly excluded due to its assumption of conditional independence between predictors, which is clearly violated in clinical data where many features are inherently correlated. More complex deep learning approaches were not implemented given the modest sample size relative to the number of features and the clear performance plateau observed across the assessed approaches.

### Model selection

Based on cross-validated performance in the training set, XGBoost was selected as the best-performing model (mean ROC AUC = 0.713, SE = 0.024), matching Random Forest performance but demonstrating superior stability (lower standard error) and the widest risk stratification (predicted probabilities ranging from 0.04 to 0.91). This model was subsequently evaluated on the held-out test set for final performance assessment.

### Variable importance and feature effects (training set)

**Fig 4:**
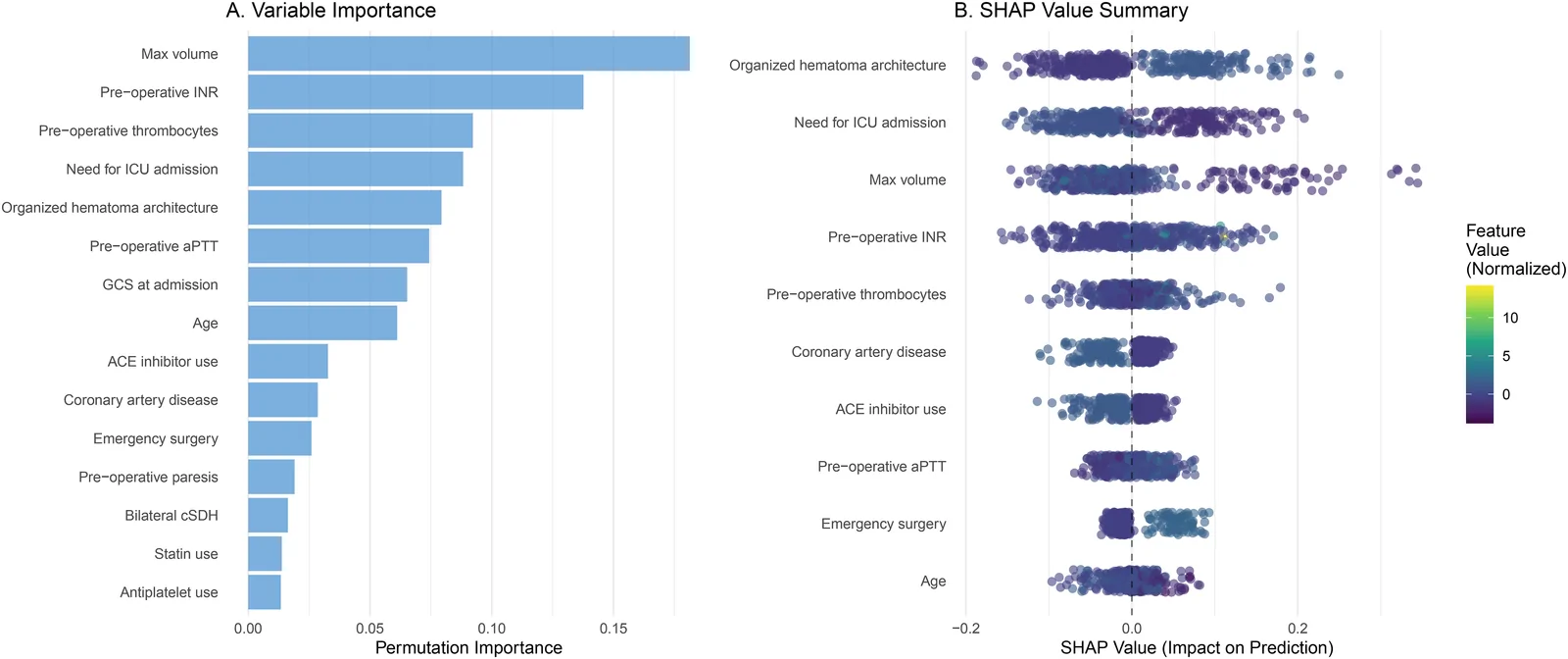
Variable importance and SHAP value analysis for xgboost recurrence prediction model. (A) Permutation-based variable importance ranking the top 15 predictors by their contribution to model discriminative performance. Importance is quantified as the decrease in model performance when each feature’s values are randomly shuffled, with higher values indicating greater predictive relevance. Maximum hematoma volume emerged as the most influential predictor (importance 0.181), followed by pre-operative coagulation parameters (INR and thrombocytes) and disease severity markers (need for ICU admission). Individual comorbidities and medications demonstrated substantially lower importance, each contributing less than 3% to model predictions. (B) SHAP (SHapley Additive exPlanations) value summary plot illustrating both the magnitude and direction of feature effects on individual patient predictions. Each point represents a single patient, with position along the x-axis indicating the feature’s impact on predicted recurrence probability (positive SHAP values increase recurrence risk, negative values decrease it). Point color represents the normalized feature value (z-score) for that variable, with blue indicating below-average values and red indicating above-average values, normalized within each variable to account for different scales across features. The vertical dashed line at zero represents no effect. Despite identifying statistically important predictors, the compressed distribution of SHAP values (predominantly between −0.2 and +0.2) demonstrates that even the most influential features shift individual predictions by less than 7 percentage points from baseline. The bidirectional effects observed for several variables (points scattered on both sides of zero) suggest complex interactions between features that vary across individual patients.

Permutation-based variable importance analysis conducted on the training set identified hematoma volume as the most influential predictor, accounting for 18.1% of the model’s discriminative capacity. Pre-operative coagulation parameters comprised the second tier of importance, with INR contributing 13.8%, platelet count 9.2%, and aPTT 7.4%. Disease severity markers, including post-operative need for ICU admission (8.8%) and GCS at admission (6.5%), formed the third tier, followed by patient age (6.1%) and having an organized hematoma type (7.9%). Individual comorbidities and medications showed substantially lower importance, with coronary artery disease, ACE inhibitor use, antiplatelet therapy, and statins each contributing less than 3% to model predictions. Variable importance analysis for the XGBoost model is presented in Fig 4A.

SHAP value analysis revealed that despite their importance rankings, the actual magnitude of feature effects was modest. The mean absolute SHAP values for the top predictors ranged from 0.069 (organized hematoma type) to 0.019 (age), indicating that even the most influential features typically shifted individual predictions by less than 7 percentage points from the baseline. The SHAP summary plot demonstrated that higher hematoma volumes consistently increased recurrence probability (red points shifted rightward), while the effects of other top predictors showed more heterogeneous patterns. Notably, ICU admission and organized hematoma type showed bidirectional effects across patients, suggesting complex interactions with other clinical features.

Partial dependence analysis revealed relatively flat relationships between most continuous predictors and recurrence probability across their typical ranges. Hematoma volume showed a gradual increase in predicted probability from approximately 0.28 at low volumes to 0.35 at high volumes, but without sharp threshold effects. INR, platelet count, aPTT, and age demonstrated minimal variation in predicted probability across their observed ranges. The GCS partial dependence plot showed an unexpected pattern, with predicted probabilities remaining stable around 0.65 for GCS scores of 3–13, then rising sharply to 0.72 for GCS scores of 14–15. This counterintuitive finding likely reflects the highly skewed distribution of GCS scores in the cohort, with 87% of patients presenting with GCS 14 or 15, and the small number of patients with lower GCS scores limiting reliable estimation of risk in that range. SHAP value analysis for the XGBoost model is presented in Fig 4B.

Examination of the actual distributions of predictor variables by outcome status revealed why these features, despite their statistical importance, provided limited discriminative capacity. Mean hematoma volume differed by only 13.5 mL between patients who developed recurrence (148.2 mL) and those who did not (134.7 mL), representing a 10% relative difference. Pre-operative INR values were nearly identical (1.15 vs 1.18), as were platelet counts (237.7 vs 244.9 per μL) and patient age (75.8 vs 74.5 years). These minimal effect sizes, while statistically detectable in a dataset of this size, fall within measurement variability and lack sufficient separation to enable confident risk stratification at the individual patient level.

### Internal validation on held-out test set

The final XGBoost model, trained on the complete training set (n = 422) using optimal hyperparameters identified through cross-validation (tree depth = 5, learning rate = 0.01, minimum node size = 5), was evaluated on the held-out test set (n = 142) to assess model generalization. The model achieved a test set ROC AUC of 0.688 (95% CI: 0.590–0.772), representing a modest decrease from the cross-validated training performance (0.713, SE = 0.024) that remained within confidence intervals, indicating minimal overfitting.

**Fig 5:**
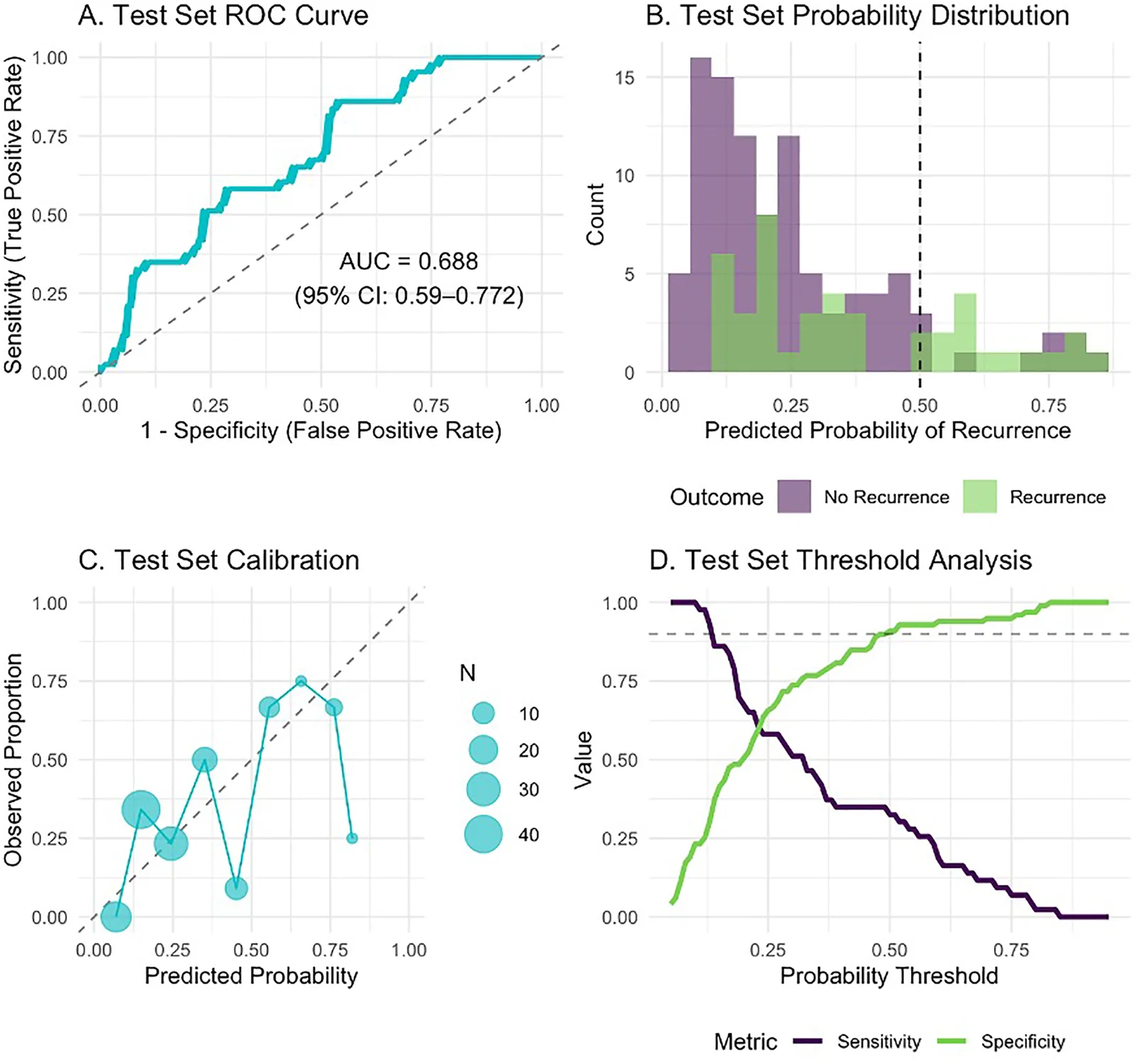
Test set performance and clinical utility analysis of XGBoost model. (A) Receiver Operating Characteristic (ROC) Curve. ROC curve for the final XGBoost model evaluated on the held-out test set (n = 142). The model achieved an area under the curve (AUC) of 0.688 (95% confidence interval: 0.590–0.772). The dashed diagonal line represents the performance of a random classifier (AUC = 0.5). (B) Distribution of Predicted Probabilities. Histogram showing the distribution of predicted recurrence probabilities stratified by actual outcome. Purple bars represent patients without recurrence (n = 99); green bars represent patients with recurrence (n = 43). The vertical dashed line indicates the default classification threshold of 0.5. Substantial overlap between outcome groups demonstrates limited discriminative capacity. (C) Calibration Plot. Agreement between predicted probabilities and observed recurrence rates across deciles of predicted risk in the test set. Points represent binned predictions, with size proportional to the number of patients in each bin (N). The dashed diagonal line represents perfect calibration. Points falling close to this line indicate good model calibration, with predicted probabilities closely matching observed outcomes. (D) Threshold Analysis. Sensitivity (purple line) and specificity (green line) as functions of the probability threshold for classifying patients as high-risk. The horizontal dashed line indicates the prespecified 90% sensitivity target. At this threshold (0.13), specificity was 30.3%, corresponding to potential imaging reduction in only 30 of 99 non-recurrence patients, insufficient for clinically meaningful risk stratification.

Test set performance metrics at the default 0.5 threshold included accuracy 73.2%, sensitivity 90.9%, and specificity 32.6%. Calibration was assessed using the calibration intercept, calibration slope, and Brier score, each with 95% confidence intervals. The calibration intercept was 0.112 (95% CI −0.291 to 0.515), indicating no significant systematic over- or under-prediction on average, consistent with the close agreement between mean predicted probability (0.285) and observed recurrence rate (0.303). The calibration slope was 0.615 (95% CI 0.268–0.962), below the ideal value of 1.0, indicating a tendency toward overconfident individual risk estimates at the extremes of the predicted probability distribution. The Brier score was 0.200 (95% CI 0.161–0.240). Risk discrimination in the test set remained modest, with mean predicted probabilities of 0.375 for patients who developed recurrence versus 0.245 for those who did not (absolute difference 0.130), consistent with training set findings (absolute difference 0.162). Performance of the final model is illustrated in Fig 5.

At the prespecified sensitivity threshold of 90%, the model achieved a test set specificity of 30.3%, corresponding to potential imaging reduction in 30 of 99 non-recurrence patients (30.3%). This finding validates the training set observation that current clinical and radiographic variables provide insufficient discriminative capacity for meaningful risk stratification. The consistency between training and test set performance confirms that the model’s limitations reflect the inherent information content of available predictors rather than overfitting to the training data. At this operating threshold (0.13), the model achieved a sensitivity of 93.0%, specificity of 30.3%, positive predictive value of 36.7%, and negative predictive value of 90.9% (95% CI 80.6−100%); 3 of 43 recurrences (7.0%) would have been missed had imaging been reduced in patients classified as low-risk. Decision curve analysis demonstrated that the model provided greater net benefit than a strategy of imaging all patients across the full range of clinically plausible threshold probabilities tested (0.05–0.50), indicating that despite its modest discrimination and calibration, model-guided surveillance would be expected to outperform uniform imaging under a range of assumptions about the relative cost of missed recurrence versus unnecessary imaging.

### Sensitivity analysis: Surgical technique as a predictor

Because surgical technique differed markedly by recurrence status in univariate analysis (Table 1), we performed an additional sensitivity analysis examining its role as a predictor. Final surgical technique, defined as the procedure performed at the time of discharge from the index admission (Methods), differed markedly by recurrence status: patients whose final technique was twist drill craniostomy recurred in 121 of 178 cases (68.0%), compared with 48 of 372 (12.9%) for burr hole craniotomy and 1 of 14 (7.1%) for bone flap craniotomy (chi-square p < 0.001). Twist drill craniostomy was also strongly associated with hematoma architecture (chi-square p < 0.001), consistent with its protocolized use in frail patients with homogeneous hematomas (Methods).

When surgical technique was added as a predictor and all three models were retrained, discrimination improved substantially: cross-validated training ROC AUC increased from 0.686 to 0.829 for logistic regression, from 0.713 to 0.849 for Random Forest, and from 0.713 to 0.833 for XGBoost. In the held-out test set, XGBoost ROC AUC increased from 0.688 (95% CI 0.590–0.772) to 0.793 (95% CI 0.713–0.876), and surgical technique became the single most influential predictor (permutation importance 0.468, exceeding hematoma volume, the next-ranked predictor, at 0.142). Clinical utility at the prespecified 90% sensitivity threshold, however, improved only modestly, from 30.3% to 34.3% specificity in the test set, indicating that the discrimination gain was driven primarily by increased confidence in classifying twist-drill-treated patients as high risk, rather than by a meaningfully expanded low-risk subgroup eligible for reduced surveillance.

Because surgical technique is itself a product of clinical judgment, protocolized in this cohort according to patient frailty and hematoma architecture rather than assigned independently of prognosis, this analysis cannot distinguish a direct technical effect of twist drill craniostomy from confounding by indication. We therefore report this analysis as a secondary, hypothesis-generating sensitivity analysis; the original models remain our primary analysis and conclusions.

### Stability of the train/test split

To assess whether performance estimates were sensitive to the particular random train/test split used, we repeated the split-and-evaluate procedure 20 times using different random seeds, refitting the final XGBoost model (fixed hyperparameters: tree depth 5, learning rate 0.01, minimum node size 5, as identified in the primary analysis) on each newly drawn training set and evaluating on the corresponding held-out test set, without re-tuning hyperparameters at each repeat, in order to isolate sampling variability in the partition from variability due to hyperparameter selection. Test set ROC AUC varied from 0.640 to 0.804 across repeats (mean 0.707, SD 0.041). The originally reported test set AUC of 0.688 fell close to this mean, indicating a representative rather than atypical draw. The mean repeated-split AUC (0.707) closely matched the cross-validated training set performance (0.713), suggesting that the model’s discriminative capacity is stable at approximately 0.70–0.71 and that the observed spread reflects sampling variability in a modest test set rather than a systematic difference between training and test performance.

### Comparison with a simplified model

To evaluate whether the added complexity of ensemble machine learning meaningfully improved prediction over a small set of established clinical and radiographic risk factors, we compared the primary XGBoost model to a simplified logistic regression model containing only six predictors: hematoma volume, hematoma architecture, postoperative ICU admission, admission GCS, preoperative INR, and emergency surgery status. This simplified model was trained and evaluated using the identical cross-validation folds and held-out test set as the primary analysis.

Within the training set, the simplified model achieved a mean cross-validated ROC AUC of 0.699 (SE 0.036). In the held-out test set, the simplified model achieved an ROC AUC of 0.766 (95% CI 0.683–0.845), numerically exceeding the primary XGBoost model (0.688, 95% CI 0.590–0.772), although the difference did not reach statistical significance (DeLong test, p = 0.056). At the prespecified 90% sensitivity threshold, the simplified model achieved 41.4% specificity in the test set (41 of 99 non-recurrence patients), compared with 30.3% for the primary model.

Unlike the primary model, in which no individual predictor showed a large or consistent effect, the simplified model’s odds ratios were substantial, with 95% confidence intervals excluding 1 for five of six predictors: postoperative ICU admission (OR 2.17, 95% CI 1.33–3.62), organized hematoma architecture relative to homogeneous architecture (OR 0.35, 95% CI 0.21–0.58), subacute architecture (OR 0.31, 95% CI 0.12–0.71), emergency surgery (OR 0.35, 95% CI 0.16–0.71), and hematoma volume (OR 1.005 per mL, 95% CI 1.001–1.009).

To assess sensitivity to the imputation method, we repeated the simplified model using multiple imputation (20 imputed datasets, predictors pooled via Rubin’s rules) in place of median/mode imputation. Pooled odds ratios were materially unchanged (postoperative ICU admission, OR 2.14, 95% CI 1.29–3.53; organized hematoma architecture, OR 0.35, 95% CI 0.21–0.58; subacute architecture, OR 0.31, 95% CI 0.13–0.73; emergency surgery, OR 0.36, 95% CI 0.17–0.75; hematoma volume, OR 1.005, 95% CI 1.001–1.009), and test-set discrimination was nearly identical (AUC 0.768, 95% CI 0.685–0.848).

These findings indicate that the additional complexity of ensemble machine learning methods and the larger predictor set used in the primary analysis did not improve, and numerically underperformed relative to, a small set of established risk factors modeled with plain logistic regression.

## Discussion

This study systematically evaluated machine learning algorithms to predict postoperative recurrence in chronic subdural hematoma using readily available clinical, demographic, laboratory, and radiographic variables. We compared three fundamentally distinct modeling approaches, regularized logistic regression, Random Forest, and XGBoost, selecting the best-performing model for final evaluation on a held-out test set. While ensemble methods demonstrated superior risk discrimination compared to linear modeling, capturing non-linear relationships and variable interactions that logistic regression could not, none of the models achieved clinically actionable risk stratification. Threshold analysis revealed that maintaining the high sensitivity required for safe de-escalation of surveillance imaging resulted in near-zero specificity across all approaches, precluding identification of a low-risk patient subgroup suitable for reduced imaging protocols. Variable importance analysis identified hematoma volume, coagulation parameters, and disease severity markers as the most influential predictors, yet the absolute effect sizes of these features remained modest. These findings suggest that currently available baseline and early postoperative clinical features possess inherent limitations in their capacity to discriminate recurrence risk, regardless of the sophistication of the modeling approach employed.

Internal validation on the held-out test set confirmed the limited clinical utility observed during model development. The selected XGBoost model achieved a test set ROC AUC of 0.688 (95% CI: 0.590–0.772), closely matching the cross-validated training performance of 0.713, demonstrating that the model’s limitations reflect the inherent information content of available predictors rather than overfitting. Calibration-in-the-large was satisfactory in the test set, with average predicted risk closely matching observed recurrence overall (calibration intercept 0.112, 95% CI −0.291 to 0.515). The calibration slope of 0.615 (95% CI 0.268–0.962), however, indicated a tendency toward overconfident individual-level predictions, a pattern consistent with the model’s modest discriminative capacity and limited test-set size; individual patient-level risk estimates from this model should therefore be interpreted cautiously. At the prespecified 90% sensitivity threshold, test set specificity was 30.3%, allowing potential imaging reduction in approximately one-third of non-recurrence patients. While this represents some discriminative capacity, the modest specificity achieved even at this threshold remains insufficient to justify implementation of risk-stratified surveillance protocols in clinical practice based on discrimination and specificity alone. Decision curve analysis offered a complementary perspective: net benefit analysis indicated that model-guided imaging decisions would outperform a uniform imaging strategy across a wide range of threshold probabilities, a finding that accounts for the relative clinical costs of missed recurrence versus unnecessary imaging in a way that specificity does not. We interpret this cautiously, as supporting formal decision-analytic evaluation of risk-stratified surveillance as a direction for future prospective research with external validation, rather than immediate practice change based on the present single-center analysis. The consistency of calibration and threshold analysis between training and test cohorts strengthens the conclusion that currently measurable preoperative and early postoperative variables cannot adequately identify a low-risk patient subgroup suitable for de-escalated surveillance imaging.

### Model performance and the predictive ceiling

Within this single-center cohort, the convergence of three fundamentally different modeling approaches to nearly identical discrimination metrics, under internal cross-validation and a held-out test set, provides evidence that the observed performance ceiling reflects the inherent predictive capacity of the measured variables in this population rather than limitations of the modeling techniques. Whether this ceiling generalizes to other institutions or case mixes cannot be determined without external validation (see Limitations). Variable importance analysis identified hematoma volume, coagulation parameters, and disease severity markers as the most influential predictors. Examination of actual effect sizes revealed why even these algorithms could not achieve clinically useful risk stratification. The differences in these variables between patients who developed recurrence and those who did not were small, often falling within typical measurement variability. SHAP analysis quantified individual feature contributions, revealing that even the most important variables had modest effects on individual predictions.

### Comparison with other machine learning studies in cSDH

While machine learning applications in chronic subdural hematoma management are emerging, existing studies address different clinical challenges. Colasurdo et al. developed a convolutional neural network for automated detection and quantification of subdural hematomas on non-contrast CT scans, achieving high sensitivity and specificity for cSDH identification [20]. Their focus on diagnostic automation differs fundamentally from recurrence prediction, serving primarily as a radiological screening tool. Fang et al. more closely aligned with our objectives by specifically predicting postoperative cSDH recurrence using a support vector machine model with combined clinical-radiomics features, reporting substantially higher accuracy and AUC than observed in our study [21]. Their approach identified history of head trauma, clinical grading scores, and midline shift parameters as key predictors. However, their relatively small sample size and lack of independent test set validation may limit generalizability and could reflect optimistic performance estimates due to overfitting.

Our study’s use of rigorous cross-validation within the training set and subsequent evaluation on a held-out test set provides more conservative and likely more realistic estimates of achievable performance with standard clinical variables. The consistency between training and test performance in our cohort suggests that the modest discriminative capacity observed represents a true ceiling for prediction using routinely collected clinical and radiographic data. A sensitivity analysis (Results) examined whether adding surgical technique, which is known prior to discharge and therefore compatible with our study’s perioperative time window, improved model discrimination. Doing so substantially increased test set AUC, from 0.688 to 0.793, driven by the markedly elevated recurrence risk associated with twist drill craniostomy in our cohort (68.0% vs. 12.9% for burr hole craniotomy), a technique reserved in our protocol for medically frail patients with homogeneous hematomas. We interpret this cautiously: because surgical technique choice is itself informed by clinical assessment of frailty and hematoma characteristics, the improved discrimination likely reflects, at least in part, the model recovering information already implicit in the treating surgeon’s judgment, rather than uncovering genuinely novel prognostic signal. Disentangling a direct technical effect of twist drill craniostomy from confounding by indication would require a study design in which technique is not determined by the same factors that predict recurrence, such as a randomized comparison or a multicenter cohort with heterogeneous technique-selection practices. We therefore retain the original predictor set for our primary analysis and conclusions, while flagging surgical technique, and specifically the drivers of twist drill craniostomy selection, as a priority for future prospective and multicenter investigation. A simplified logistic regression model containing only six established risk factors performed comparably to, and numerically better than, our primary ensemble model (test set AUC 0.766 versus 0.688; Results), with a larger proportion of non-recurrence patients potentially eligible for reduced surveillance at the same sensitivity threshold (41.4% versus 30.3%). This finding reinforces our central conclusion: the discriminative ceiling observed across modeling approaches reflects the inherent information content of routinely available clinical and radiographic variables rather than a limitation of any specific algorithm, including the ensemble methods that form our primary analysis. It also suggests that a small set of interpretable, readily available variables captures most of the currently achievable predictive signal for cSDH recurrence, without need for more complex modeling. To contextualize our AUC of 0.688 against traditional, non-machine-learning cSDH recurrence-risk scores, a systematic external validation of published prognostic models, including the Danish cSDH recurrence models [12], found externally validated discrimination ranging from below chance level to a concordance index of 0.65 [23], comparable to or lower than the discrimination achieved by our primary and simplified models in the present study. This suggests that the modest discrimination we observed is consistent with, rather than uniquely worse than, the ceiling reported elsewhere in the cSDH recurrence-prediction literature.

### Biological and clinical implications

The convergence of results across modeling approaches suggests that recurrence may be largely determined by factors not captured in routine clinical assessment. Potential unmeasured determinants include molecular and genetic factors influencing inflammation and angiogenesis, microscopic characteristics of the hematoma membrane, and stochastic biological processes that are inherently unpredictable.

These findings have important implications for postoperative surveillance strategies. Our inability to identify a low-risk patient subgroup using comprehensive clinical data and machine learning algorithms, confirmed through internal validation, provides justification for maintaining uniform surveillance practices in all patients. However, this raises the fundamental question of whether routine imaging-based surveillance is the optimal approach. Schucht et al. conducted a randomized controlled trial comparing routine follow-up CT imaging versus symptom-driven imaging, demonstrating no benefit of routine surveillance on clinical outcomes [11]. Their findings showed that routine imaging resulted in more reoperations and higher costs without improving patient outcomes. These results suggest that rather than attempting to refine imaging-based risk stratification protocols using machine learning, a symptom-driven approach to postoperative surveillance may be more clinically appropriate and cost-effective. Our recurrence rate of 30.1% is at the upper end of the previously reported 5–33% range [9] and likely reflects our institution’s intensive post-discharge imaging protocol, which continued until radiological resolution was documented. More frequent surveillance imaging is likely to detect a greater number of asymptomatic or early recurrences than a symptom-driven approach would, while our definition, requiring reoperation, simultaneously excludes radiographically evident recurrences that were managed conservatively, most notably small, asymptomatic residual collections in patients with pre-existing brain atrophy, which were considered resolved without further intervention. These two features of our protocol act in partially offsetting directions, and the net effect on our observed recurrence rate relative to centers using symptom-driven surveillance cannot be determined from our data alone. Routine post-discharge imaging was standard practice throughout most of our study period (2015–2023), predating the 2019 publication of Schucht et al.’s randomized trial questioning its benefit [11]. Our findings speak to whether risk-stratified imaging reduction is achievable within this established surveillance framework, rather than constituting a retrospective endorsement of routine imaging; how our model or a symptom-driven protocol would perform in a prospectively symptom-driven surveillance cohort cannot be determined from the present retrospective, imaging-driven dataset and would require a prospective study design. Because the model incorporates postoperative variables, it is intended for application at discharge rather than at the time of the initial surgical decision, using information that is already known to the clinical team by that point. This distinguishes it from a preoperative counseling tool and situates it specifically as a discharge-planning aid for surveillance intensity, the clinical use case that motivated this study. Anticoagulation management was not static over the study period. The proportion of patients treated with vitamin K antagonists (Marcumar) fell from 18.4% (78/424) of patients admitted in 2015–2021 to 4.3% (6/138) in 2022–2023, consistent with the broader international shift toward direct oral anticoagulants over this period. Within our cohort, Marcumar-treated patients had significantly higher recurrence rates than those treated with direct oral anticoagulants (33.3% vs. 17.5%, chi-square p = 0.017). Because anticoagulated patients represented a minority of the overall cohort (13.4–23.9% depending on period), this shift can account for only a small fraction of the substantially larger difference in overall recurrence rate observed between the two periods (see Limitations), and the remaining difference is not explained by any practice change we identified. No patients in our cohort received middle meningeal artery embolization (MMAE), an adjunctive treatment increasingly used to reduce cSDH recurrence risk that has gained substantial adoption following supportive randomized trial evidence. Our findings therefore characterize recurrence prediction in a surgery-only treatment paradigm and may not generalize to contemporary practice settings where MMAE is routinely offered, particularly to patients at elevated recurrence risk, which could itself alter the achievable discriminative ceiling by shifting recurrence risk in ways not captured by our model.

### Future directions

Future research aimed at improving recurrence prediction will likely require identification of novel predictive features beyond standard clinical variables. Promising avenues include advanced radiomics analysis to extract subtle quantitative imaging features not appreciated by visual inspection, molecular profiling of hematoma fluid or membrane tissue to identify inflammatory or angiogenic biomarkers, and genomic or proteomic markers of individual susceptibility to recurrence. Alternatively, the consistent finding across multiple studies that recurrence prediction remains challenging may indicate that prevention strategies, such as optimization of surgical technique or pharmacological interventions targeting hematoma membrane biology, represent more promising approaches than risk stratification alone.

External validation in independent cohorts from other institutions will be essential to confirm whether the performance ceiling observed in our study represents a generalizable limitation or whether institution-specific factors influence recurrence prediction. Multi-center collaborations with larger sample sizes may also enable identification of rare but highly predictive features that cannot be detected in single-center studies. Future studies would also benefit from capturing precise recurrence and mortality dates to enable time-to-event modeling, which would more appropriately account for variable follow-up duration and the competing risk of death in this elderly population than the binary outcome definition used here. Given the significant recurrence-rate difference we observed between vitamin K antagonists and direct oral anticoagulants (Discussion), future models may also benefit from distinguishing anticoagulant subtype rather than combining all oral anticoagulation into a single category.

### Limitations

This single-center, retrospective study has several limitations. First, we lacked external validation, so model performance and calibration may not generalize to other institutions, with different case mixes, and / or follow-up strategies; larger, ideally multicenter, validation cohorts will be needed to obtain more precise and generalizable performance estimates. Our held-out test set, though stratified to preserve outcome prevalence, contained only 43 recurrence events among 142 patients; formal sample size guidance for external validation of clinical prediction models indicates that substantially more events are typically required to precisely estimate discrimination and calibration [22], and the width of our reported confidence intervals (e.g., test set ROC AUC 95% CI 0.590–0.772) reflects this constraint. Repeated random splitting confirmed considerable variation in test set AUC (0.640–0.804) depending on the specific patients allocated to the held-out set, reinforcing that our single reported test set estimate should be interpreted alongside its confidence interval and the cross-validated training performance rather than in isolation. Second, selection and information bias are possible because 10.5% of the initial cohort lacked outcome data, and some predictors required imputation, while radiographic measurements and architecture classification are subject to interobserver and software variability, although excluded patients did not show elevated mortality relative to included patients (Results). Architecture classification was performed with blinding to outcome and demonstrated high interrater reliability in a related cohort [10], but blinding status was not separately documented for software-assisted volumetric and midline shift measurements. Bilateral hematomas were characterized using maximal unilateral dimensions alongside total bilateral volume rather than fully independent per-side measurements, which may lose laterality-specific information relevant to recurrence. Missing predictor values were handled using median/mode imputation rather than multiple imputation; given the low overall missingness (maximum 4.8% for any single variable), we expected minimal practical difference between the two approaches and confirmed this directly for a simplified predictor set (Results), where multiple imputation yielded materially unchanged odds ratios and test-set discrimination. Third, the outcome definition relied on reoperation for radiographic or clinical recurrence, which may reflect local practice patterns and thresholds for revision, including our routine post-discharge imaging protocol, and did not capture radiographically evident recurrences managed conservatively. Fourth, an attempted temporal validation (training on patients admitted 2015−2021, testing on 2022−2023) revealed a substantially lower observed recurrence rate in the 2022−2023 subgroup (3.6% vs. 38.7%) that could not be fully explained: a shift toward direct oral anticoagulants, associated with lower recurrence than vitamin K antagonists in our cohort, accounted for only a small part of this difference, and differential patient exclusion was not a contributing factor. We additionally identified a marked reduction in outcome data completeness for patients admitted in 2020 (87% missing outcome data, versus less than 7% in all other years), coinciding with the COVID-19 pandemic. Because of the resulting small number of events in the temporal test period, formal temporal validation could not be reliably performed, and sensitivity analyses using alternative recurrence-definition windows (e.g., 3 or 12 months), as well as time-to-event and competing-risk analyses accounting for death before recurrence, were not possible, as these would require a time-to-recurrence variable not available in structured form in our dataset. Finally, despite cross-validation and a held-out test set, residual overfitting cannot be fully excluded, and no patients in our cohort received middle meningeal artery embolization, an adjunctive treatment increasingly used to reduce recurrence risk, which may limit generalizability to contemporary practice settings.

## Conclusion

In this single-center study, using internal cross-validation and a held-out test set, machine learning algorithms did not achieve clinically actionable prediction of postoperative recurrence in chronic subdural hematoma using routinely available clinical, laboratory, and radiographic data. External validation in independent cohorts is required to determine whether this finding generalizes beyond our institution. Decision curve analysis nonetheless suggested that model-guided imaging decisions could outperform uniform surveillance across a range of plausible clinical assumptions, a hypothesis-generating finding meriting prospective evaluation. Despite employing diverse modeling paradigms, all approaches converged to a similar performance ceiling, indicating that the limitation lies in the intrinsic information content of the variables rather than in algorithmic sophistication. This highlights that recurrence is likely driven by biological, technical, or stochastic factors not captured in standard datasets. These findings support the continued practice of uniform postoperative vigilance while emphasizing that meaningful advances in risk prediction will require integration of novel biomarkers, radiomic features, or intraoperative metrics.

## Supporting information

S1 Table Hyperparameter tuning grids. For each model, hyperparameters were tuned via a regular grid search (tidymodels::grid_regular()), which generates evenly spaced candidate values across the stated range for each parameter, evaluated in all combinations via 10-fold stratified cross-validation on the training set (n = 422). Parameters using a log10-transformed range (penalty, learning rate) are evenly spaced in log10 space before being converted back to their natural scale.
(DOCX)

S2 Table Regularized logistic regression — detailed training-set performance. (DOCX)

S3 Table Random Forest — detailed training-set performance. (DOCX)

S1 Fig Per-model predicted-probability distributions and threshold curves. (A) Logistic regression: distribution of predicted probabilities of recurrence, by observed outcome (10-fold cross-validation, training set). Dashed line indicates the default 0.5 classification threshold. (B) Logistic regression: sensitivity (teal) and specificity (red) as a function of probability threshold; dashed horizontal line marks the 90% sensitivity target used elsewhere in the manuscript. (C) Random Forest: distribution of predicted probabilities, as in panel A. (D) Random Forest: threshold analysis, as in panel B. Corresponds to main text Table S2 (logistic regression) and Table S3 (Random Forest); compare with main text Fig. 3 (XGBoost, the primary model).
(DOCX)

## Abbreviations

ACE: Angiotensin-Converting Enzyme
aPTT: Activated Partial Thromboplastin Time
ASA: American Society of Anesthesiologists
AUC: Area Under the Receiver Operating Characteristic Curve
CT: Computed Tomography
cSDH: , Chronic Subdural Hematoma
DOAC: Direct Oral Anticoagulant
DRKS: Deutsches Register Klinischer Studien (German Clinical Trials Register)
EK: Ethikkommission (Ethics Committee)
GCS: Glasgow Coma Scale
ICU: Intensive Care Unit
INR: International Normalized Ratio
IQR: Interquartile Range;
LASSO: Least Absolute Shrinkage and Selection Operator
NIHSS: National Institutes of Health Stroke Scale
PACS: Picture Archiving and Communication System
ROC: Receiver Operating Characteristic
SDH: Subdural Hematoma
SHAP: SHapley Additive exPlanations
STROBE: Strengthening the Reporting of Observational Studies in Epidemiology
XGBoost: Extreme Gradient Boosting
