# Supplementary material for: Classification of recurrence status after surgical treatment of chronic subdural hemorrhage – A machine learning approach
Source: PLoS One. 2026 Sep 18;21(9):e0346756. doi: 10.1371/journal.pone.0346756 (PMC13588510; doi:10.1371/journal.pone.0346756)
Supplement: Supplementary file 1 [file pone.0346756.s001.docx]

# **S1 Table. Hyperparameter tuning grids**

For each model, hyperparameters were tuned via a regular grid search (tidymodels::grid_regular()), which generates evenly spaced candidate values across the stated range for each parameter, evaluated in all combinations via 10-fold stratified cross-validation on the training set (n=422). Parameters using a log10-transformed range (penalty, learning rate) are evenly spaced in log10 space before being converted back to their natural scale.

**S1a. Regularized logistic regression (elastic net) — 50 combinations**

| **Hyperparameter** | **Range tested** | **Levels** | **Selected value** |
| --- | --- | --- | --- |
| Penalty (λ) | 10⁻⁴ to 10⁻⁰·⁵ (0.0001–0.316) | 10 | 0.053 |
| Mixture (α) | 0 (ridge) to 1 (lasso) | 5 | 0.75 |

*α = 0 corresponds to pure ridge regression, α = 1 to pure lasso; intermediate values interpolate between the two penalty types.*

**S1b. Random Forest — 30 combinations**

| **Hyperparameter** | **Range tested** | **Levels** | **Selected value** |
| --- | --- | --- | --- |
| mtry (predictors per split) | 2 to 20 | 6 | 12 |
| min_n (minimum node size) | 2 to 40 | 5 | 11 |

*Number of trees was fixed at 500 (not tuned). Variable importance was computed via permutation importance on the final model.*

**S1c. XGBoost — 36 combinations**

| **Hyperparameter** | **Range tested** | **Levels** | **Selected value** |
| --- | --- | --- | --- |
| tree_depth (maximum tree depth) | 3 to 8 | 3 | 5 |
| learn_rate (step-size shrinkage) | 0.01 to 0.1 | 4 | 0.01 |
| min_n (minimum node size) | 5 to 20 | 3 | 5 |

*Number of boosting rounds was fixed at 500 (not tuned). Objective: binary:logistic; evaluation metric: AUC.*
