# Supplementary material for: Classification of recurrence status after surgical treatment of chronic subdural hemorrhage – A machine learning approach
Source: PLoS One. 2026 Sep 18;21(9):e0346756. doi: 10.1371/journal.pone.0346756 (PMC13588510; doi:10.1371/journal.pone.0346756)
Supplement: Supplementary file 2 [file pone.0346756.s002.docx]

# **S2 Table. Regularized logistic regression — detailed training-set performance**

| **Metric** | **Value** |
| --- | --- |
| Optimal penalty (λ) | 0.053 |
| Optimal mixture (α) | 0.75 |
| Cross-validated ROC AUC (SE) | 0.686 (0.036) |
| Predicted probability range | 0.14 – 0.46 |
| Mean predicted probability, recurrence | 0.333 |
| Mean predicted probability, no recurrence | 0.287 |
| Absolute risk separation | 0.046 |
| Specificity at 85% sensitivity threshold | ~0% |
| Specificity at 90% sensitivity threshold | ~0% |
| Specificity at 95% sensitivity threshold | ~0% |
| Sensitivity at 0.5 default threshold | not clinically actionable (see note) |

*At the default 0.5 threshold, the model's predicted probabilities were too compressed (range 0.14–0.46) for a conventional confusion matrix at this threshold to be clinically meaningful; see main text Fig. 2 for the full ROC curve.*

**Narrative summary**

We first developed a regularized logistic regression model using elastic net penalization to handle correlated predictors and perform automatic feature selection. Hyperparameter tuning across 50 combinations of penalty strength and mixing parameters (Table S1a) identified the optimal configuration shown above, favoring lasso-type regularization with substantial feature selection.

Examination of predicted probabilities revealed substantial compression of risk estimates, with all predictions ranging between 0.14 and 0.46. While the difference in mean predicted probability between patients with and without recurrence was statistically discernible and contributed to the ROC AUC of 0.686, the absolute separation between risk groups was insufficient for clinical risk stratification.

At all examined sensitivity thresholds (85%, 90%, 95%), the corresponding specificity approached zero, indicating that the model could not identify any patients as low-risk without unacceptably compromising recurrence detection. These findings suggest that the available baseline and early postoperative clinical variables, when combined in a linear fashion through regularized logistic regression, provide limited ability to discriminate between patients who will and will not develop recurrence.
