# Supplementary material for: Classification of recurrence status after surgical treatment of chronic subdural hemorrhage – A machine learning approach
Source: PLoS One. 2026 Sep 18;21(9):e0346756. doi: 10.1371/journal.pone.0346756 (PMC13588510; doi:10.1371/journal.pone.0346756)
Supplement: Supplementary file 3 [file pone.0346756.s003.docx]

# **S3 Table. Random Forest — detailed training-set performance**

| **Metric** | **Value** |
| --- | --- |
| Optimal mtry | 12 |
| Optimal min_n | 11 |
| Number of trees | 500 |
| Cross-validated ROC AUC (SE) | 0.713 (0.028) |
| Delta AUC vs. logistic regression | +0.027 |
| Predicted probability range | 0.04 – 0.83 |
| Mean predicted probability, recurrence | 0.387 |
| Mean predicted probability, no recurrence | 0.276 |
| Absolute risk separation | 0.111 |
| Sensitivity at 0.5 threshold | 22% |
| Specificity at 0.5 threshold | 93% |
| True negatives / total no-recurrence (0.5 threshold) | 274 / 295 |
| True positives / total recurrence (0.5 threshold) | 28 / 127 |
| Specificity at 85% sensitivity threshold | ~0% |
| Specificity at 90% sensitivity threshold | ~0% |
| Specificity at 95% sensitivity threshold | ~0% |

**Narrative summary**

To explore whether non-linear relationships and feature interactions could improve predictive performance, we developed a Random Forest model with 500 trees. Hyperparameter tuning across 30 combinations of predictor sampling and minimum node size (Table S1b) identified the optimal configuration shown above.

The model demonstrated superior risk stratification compared to logistic regression, with a wider predicted-probability range (0.04–0.83 vs. 0.14–0.46) and greater absolute risk separation (0.111 vs. 0.046), indicating that the Random Forest algorithm successfully identified non-linear patterns and variable interactions that linear modeling could not capture.

Despite these improvements in discrimination metrics, threshold analysis revealed that the enhanced predictive performance did not translate into clinically actionable risk stratification for the intended application of reducing surveillance imaging. When prioritizing high sensitivity as required for safe de-escalation of surveillance, the model's specificity collapsed to near zero at all examined sensitivity thresholds (85%, 90%, 95%), mirroring the findings with logistic regression and suggesting that the limitation lies not in the modeling approach but in the inherent predictive capacity of the available clinical and radiographic features.
