# Supplementary material for: Classification of recurrence status after surgical treatment of chronic subdural hemorrhage – A machine learning approach
Source: PLoS One. 2026 Sep 18;21(9):e0346756. doi: 10.1371/journal.pone.0346756 (PMC13588510; doi:10.1371/journal.pone.0346756)
Supplement: Supplementary file 4 [file pone.0346756.s004.docx]

# **S1 Figure. Per-model predicted-probability distributions and threshold curves**

*(A) Logistic regression: distribution of predicted probabilities of recurrence, by observed outcome (10-fold cross-validation, training set). Dashed line indicates the default 0.5 classification threshold. (B) Logistic regression: sensitivity (teal) and specificity (red) as a function of probability threshold; dashed horizontal line marks the 90% sensitivity target used elsewhere in the manuscript. (C) Random Forest: distribution of predicted probabilities, as in panel A. (D) Random Forest: threshold analysis, as in panel B. Corresponds to main text Table S2 (logistic regression) and Table S3 (Random Forest); compare with main text Fig. 3 (XGBoost, the primary model).*
